# Crystal structure of L-aspartate aminotransferase from *Schizosaccharomyces pombe*

**DOI:** 10.1371/journal.pone.0221975

**Published:** 2019-08-29

**Authors:** Soo Yeon Jeong, Hyeonseok Jin, Jeong Ho Chang

**Affiliations:** 1 Department of Biology Education, Kyungpook National University, Daegu, Republic of Korea; 2 Research Institute for Phylogenomics and Evolution, Kyungpook National University, Daegu, Republic of Korea; Victoria University of Wellington, NEW ZEALAND

## Abstract

L-aspartate aminotransferase is a pyridoxal 5ʹ-phosphate-dependent transaminase that catalyzes reversible transfer of an α-amino group from aspartate to α-ketoglutarate or from glutamate to oxaloacetate. L-aspartate aminotransferase not only mediates amino acid and carbohydrate metabolism but also regulates the cellular level of amino acids by catalyzing amino acid degradation and biosynthesis. To expand our structural information, we determined the crystal structure of L-aspartate aminotransferase from *Schizosaccharomyces pombe* at 2.1 Å resolution. A structural comparison between two yeast L-aspartate aminotransferases revealed conserved enzymatic mechanism mediated by the open–closed conformational change. Compared with higher eukaryotic species, L-aspartate aminotransferases showed distinguishable inter-subunit interaction between the N-terminal arm and a large domain of the opposite subunit. Interestingly, structural homology search showed varied conformation of the N-terminal arm among 71 structures of the family. Therefore, we classified pyridoxal 5ʹ-phosphate-dependent enzymes into eight subclasses based on the structural feature of N-terminal arms. In addition, structure and sequence comparisons showed strong relationships among the eight subclasses. Our results may provide insights into structure-based evolutionary aspects of pyridoxal 5ʹ-phosphate-dependent enzymes.

## Introduction

L-aspartate aminotransferase (AST, EC 2.6.1.1) is not only a key metabolic enzyme that links amino acid metabolism to carbohydrate metabolism through reversible transamination reaction but also an enzyme that regulates the cellular level of amino acid by catalyzing amino acid degradation and biosynthesis [1, 2]. The enzyme catalyzes a reversible reaction using pyridoxal 5ʹ-phosphate (PLP) as a cofactor:

L−aspartate+2−oxoglutarate⇔oxaloacetate+L−glutamate

AST enzyme is highly conserved across species, owing to its essential role in varied metabolic pathways. ASTs are found from bacterial to eukaryotic species, with sequence identity ranging from 20%–80% [3]. AST from *Saccharomyces pombe* shares relatively high sequence identity with those of chicken cytosolic (46%), *Saccharomyces cerevisiae* (49%), and *Escherichia coli* (37%). On the other hand, *S*. *pombe* AST shares relatively low sequence identity with those of *Pyrococcus horikoshii* (16%), *Thermus thermophilus* (17%), and *Thermotoga maritima* (15%). The structure of cytosolic or mitochondrial AST from chicken [4–7], pig heart [8], *Saccharomyces cerevisiae* [9], and *Escherichia coli* [10, 11] have been reported not only in its apo form but also as a complex with its cofactor PLP, pyridoxamine-5'-phosphate (PMP), or dicarboxylic inhibitor maleate [12–14]. Although there are variations in sequence identities, the overall domain folds of ASTs are highly similar. A detailed enzymatic mechanism was also proposed based on previously reported structures [5, 12, 13].

Among three families (α, β, and γ) of PLP-dependent enzymes, the aminotransferases were classified as an α-family according to the characteristics of their catalytic reaction and sequence similarity [15]. Thus, Goldsmith et al. subsequently classified the PLP-dependent enzymes based on their fold types and sequence variations, i.e., five different fold patterns were identified as fold types I to V [16]. Lindqvist group provided additional classifications for the family of PLP-dependent enzyme according to their structural similarities [17, 18]. They suggested six PLP-dependent enzyme subclasses based on multiple structure alignments. The major difference between the subclasses were observed for the N-terminal region of the subunit which is involved in substrate binding and inter-subunit interactions. The variation of N-terminal region within subclasses correlates to their functions with regard to oligomerization and catalytic activity.

AST generally forms a homodimer consisting of two active sites in the vicinity of subunit interfaces; these active sites bind to its cofactor PLP and substrate independently. Each subunit is composed of three parts: large domain, small domain, and N-terminal arm. The active site is situated in the cavity formed by two large domains and one small domain. Conformational transition of the small domain was observed in accordance with the binding of dicarboxylic inhibitors [5, 12, 19]. For example, the structures of chicken mitochondrial ASTs showed that the small domains of each subunit rotated toward the large domains of the other subunit, thereby burying the active site by closing the cavity upon recognition of a substrate or inhibitor [19].

In this study, to suggest a broader view of the three-dimensional structure and show the structural features of PLP-family proteins, we determined the crystal structure of AST from *Schizosaccharomyces pombe*. Based on the structural analysis of *Sp*AST and other homologues, distinguishable inter-subunit interaction was observed between the N-terminal arm and the large domain of the subunit opposite to the N-terminal arm. In addition, the conformation of the N-terminal arm of PLP-dependent enzymes can be categorized into eight types via structural homology search. This study may provide insights into structural and evolutionary relationships for a broad spectrum of ASTs.

## Materials and methods

### Protein expression and purification

The gene encoding *Schizosaccharomyces pombe* L-aspartate aminotransferase (*Sp*AST, Uniprot ID O42652) was amplified from its genomic DNA and cloned into pET26b. An accidental point mutation D153E was found in the recombinant DNA. However, the mutation was unlikely affect the enzymatic function due to its location as far from the active site or dimeric interface. The plasmid was transformed into BL21 (DE3) star, and cells were grown in LB medium. The recombinant *Sp*AST protein was induced by 0.3 mM isopropyl-β-D-thiogalactopyranoside at A_600_ of 0.6, and cells were further incubated at 20°C for 18 h. Cells were then collected and lysed by sonication; cell debris was then precipitated by centrifugation. The supernatant was incubated with Ni^+^-NTA resin for 90 min, and the C-terminal His_6_-tagged *Sp*AST protein was eluted by 250 mM imidazole after being washed with 5 mM imidazole. Samples were further purified using Hiprep 16/60 sephacryl S300 column (GE healthcare), which was equilibrated with 20 mM Tris pH 7.5, 150 mM NaCl, and 2 mM DTT. Proteins were concentrated to 24 mg/mL and stored at −80°C until further use.

### Crystallization and X-ray diffraction data collection

Crystallization of *Sp*AST protein was performed by employing hanging drop vapor diffusion method at 20°C. The crystal was observed at a concentration of 24 mg/mL protein in 20% (w/v) polyethyleneglycol 8000 and 0.1 M sodium HEPES (pH 8.5). Crystals were then cryoprotected using a crystallization solution containing 21% (v/v) glycerol and flash frozen in liquid nitrogen. The crystal was diffracted and collected at 100 K at 0.9795 Å on beamline 5C at the Pohang Accelerator Laboratory (PAL, Republic of Korea), using a Pilatus-3 6M detector (Switzerland) [20]. Data was further processed using the HKL2000 package [21]. The data was scaled in the P2_1_ space group.

### Structure determination

The structure was determined by molecular replacement method using AutoMR software of the PHENIX software package with AST from *Saccharomyces cerevisiae* AST (PDB code, 1YAA) as a search model [22, 23]. The initial model was obtained by autobuild in the PHENIX package [24] and then manually built using the program COOT [25]. The model was refined by phenix.refine in the PHENIX package [22], using XYZ coordinate, rigid body, individual B-factors, TLS parameters, and simulated annealing with 5% reflections for test sets. Detailed data collection and refinement statistics are listed in Table 1.

**Table 1:** Statistics for data collection and refinement.

	*Sp*AST
**Data collection**	
Space group	P2_1_
Cell dimensions	
*a*, *b*, *c* (Å)	62.1, 53.1, 130.3
*α*, *β*, *γ* (°)	90, 96.7, 90
Resolution (Å)	50–2.1 (2.18–2.1)^*a*^
Measured reflections	249972
Unique reflections	49395
Completeness (%)	99.8 (100)
Average (*I*/σ)	31.4 (18.8)
*R*_merge_ (%)	7.5 (14.3)
Redundancy	5.1 (5.2)
**Refinement**	
Resolution (Å)	39.3–2.1
No. of reflections	49365
R_*work*_/R_*free*_ (%)^*b*^	14.3/18.3
No. of atoms	
Protein	6462
Ligands	22
Water	727
Root mean square deviations	
Bond lengths (Å)	0.007
Bond angles (°)	0.817
B-factors	
Protein	17.2
Ligands	24.8
Water	27.4
Clash score	2.74
Ramachandran plot	
Most favored (%)	97.7
Allowed (%)	2.1
Outlier (%)	0.2

^*a*^ Values in parentheses are for the highest shell.

^*b*^ *R*_free_ was calculated using 5% of the reflections for test sets.

### Homology search and phylogenetic analysis

The homologous proteins were searched by aligning their structures to that of *Sp*AST from DALI server [26]. The subclasses of the proteins were classified by careful structural comparison especially focused on the N-terminal region of the proteins. Evolutionary relationships between the subclasses were further analysed among 29 homologues by sequence alignment followed by construction of phylogenetic tree using ClustalX [27] and Mega7 [28] softwares, respectively. The phylogenetic tree was constructed by Maximum Likelihood (ML) method and bootstrapping 100 replications using WAG +I +G model.

## Results and discussion

### Overall structure of *Sp*AST

**Fig 1:**
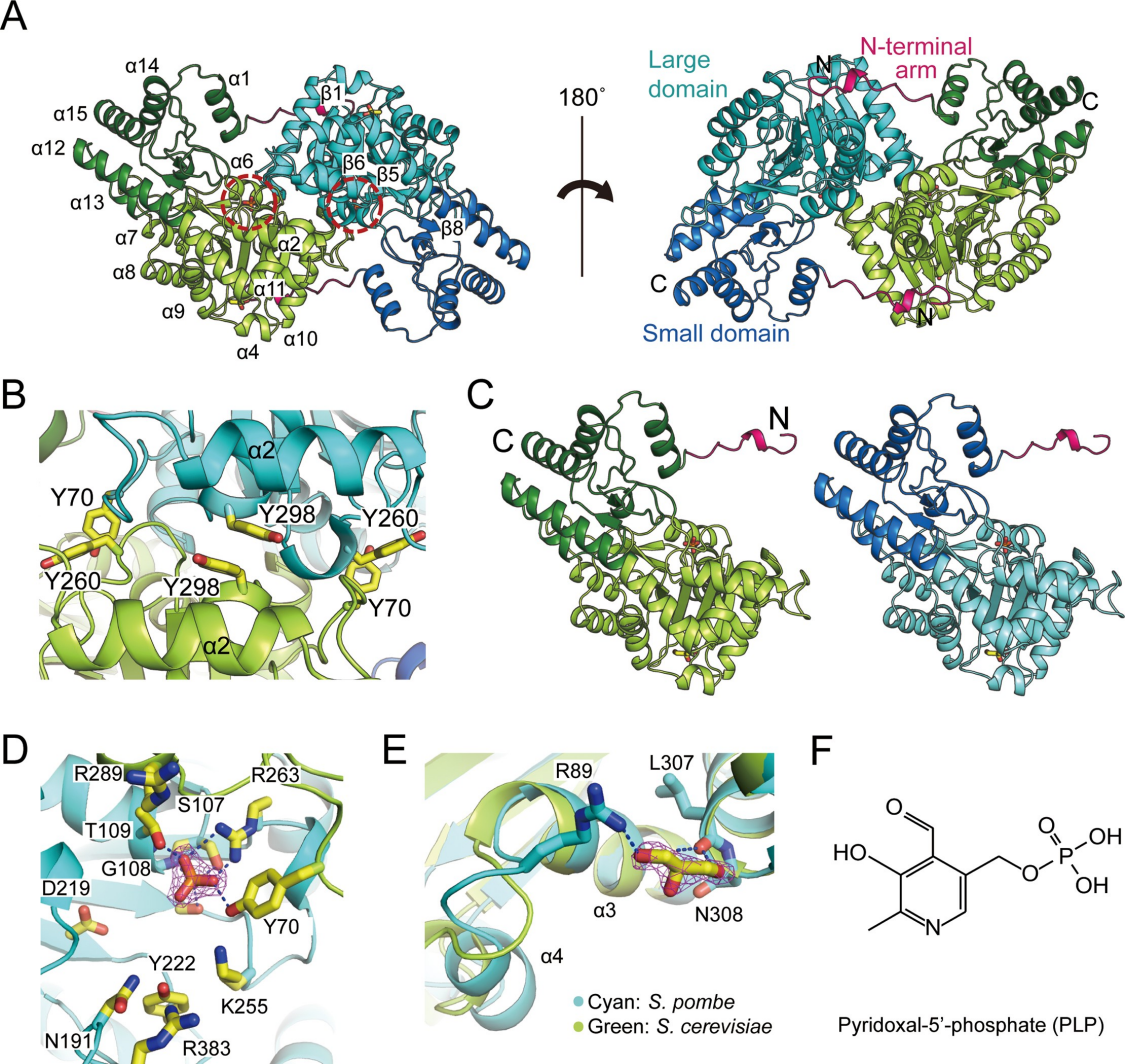
Overall structure of *Sp*AST. (A) The homodimeric crystal structure of *Sp*AST. The small domains (green and blue), large domains (light green and cyan), and N-terminal arms (hot pink) are presented. The phosphates in the active site and glycerol molecules are represented as yellow stick. The active sites are highlighted as dashed red circles. (B) Pi interactions at the subunit interface. Pairs of tyrosine residues, namely Y298–Y298 at helix α2 as well as Y70–Y298 at loop α2–α3 and helix α11, respectively, are represented as yellow stick. (C) Two monomers of the homodimeric structure are represented in the same orientation and color as given in Fig 1A. 2mFo-DFc map for the (D) phosphate as well as for the (E) glycerol molecule. (F) The chemical structures of pyridoxal 5ʹ-phosphate is shown.

The structure of *Sp*AST forms a very stable homodimer with an unusually extended interface (Fig 1A). The solvent accessible area of subunit interface was calculated as 3004.5 Å (16.7% of total solvent accessible area) from PISA analysis, and 85 residues (20.8% of total residues) were located at the interface [29]. Each subunit of *Sp*AST folds up into three spatially and functionally distinct regions: a large domain, a small domain, and the N-terminal arm. The large domain (residues 48–322) has a central beta sheet, which encompasses 47 residues across seven strands surrounded by alpha helices, thereby resembling Rossmann fold. The small domain (residues 16–47 and 323–409) contains two antiparallel beta strands and is surrounded by five helices. Two domains are linked with each other through a long alpha helix α12. Fifteen amino acids present in the N-terminal extended from α1 of the small domain and formed a loop. This loop wraps across the subunit and interacts with the loop α9–β6, helix α10, and loop β7–α10 of the large domain.

The subunits of the homodimer are tightly associated mainly with their large domains through the salt bridges and hydrogen bonds present between side chains as well as between the main chain and the side chain. The water molecules also significantly contribute to inter-subunit interaction. Also, many hydrophobic residues are aligned at the interface contributing hydrophobic interaction between two molecules of dimer. Among 85 residues involved in the dimeric interaction, 7 residues formed salt bridges, 28 residues formed hydrogen bond, and 40 residues contributed hydrophobic interactions. Two tyrosine residues in both of the large domains, Tyr298 in helix α11, forms a face-to-face pi interaction with each other within a distance of 4 Å, contributing to stable dimerization of the dimer. Tyr70 and Tyr260 of both the large domains are also in close distance within 3.6 Å with a nearly perpendicular shape (Fig 1B). This also might form a T-shaped interaction, which renders stability not only for dimerization but also for a local conformation of the active site. The root mean square deviation (rmsd) of two subunits of a dimer is 0.26 Å, which indicates that there are little differences between two subunits (Fig 1C).

Unexpectedly, two additional electron densities were found at the active site and the surface that could be fitted to phosphate and glycerol molecules (Fig 1D and 1E). Notably, the loop α3–α4, and helix α4 showed an altered conformation in comparison with the conformation of the previously reported structures [7, 9]. The conformational transition might be likely owing to the binding of glycerol molecule, which indicates that the transition could be the result of an artifact during the crystallization process in AST–glycerol complex formation.

### Active site

**Fig 2:**
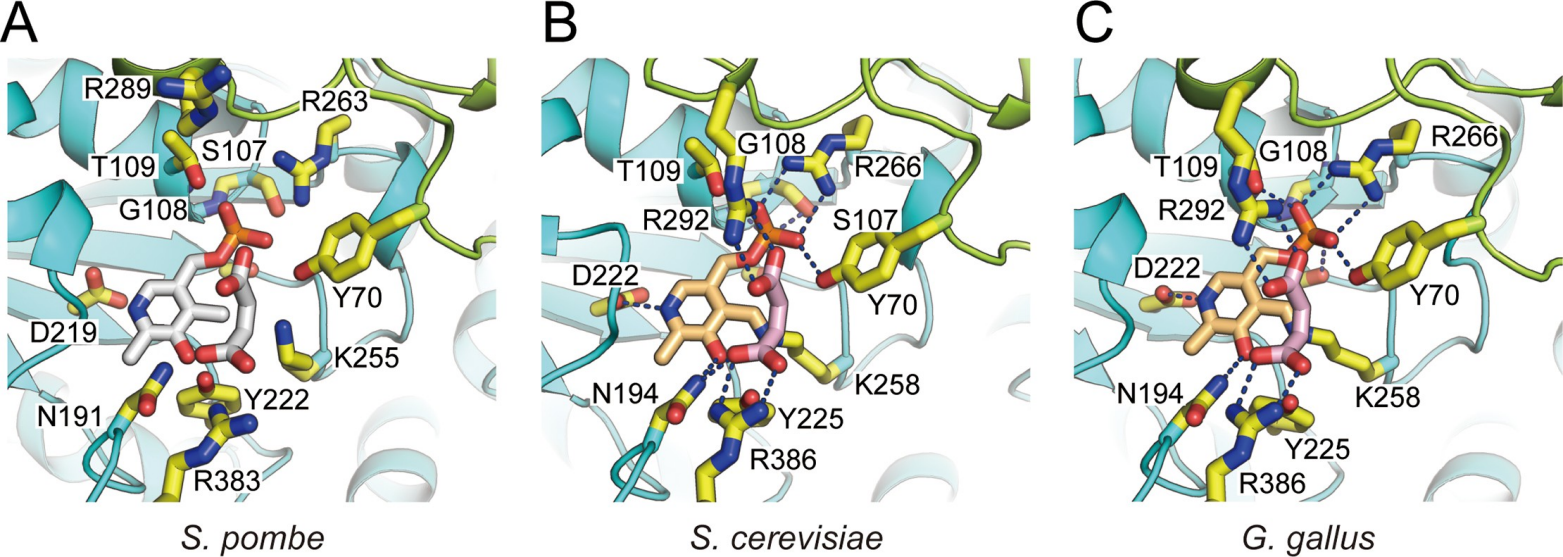
Comparison of active sites. Three active sites of AST from (A) *S*. *pombe*, (B) *S*. *cerevisiae* (PDB code, 1YAA), and (C) *G*. *gallus* (PDB code, 2CST) are represented. For the structure derived from *S*. *pombe*, the inhibitor maleate and the cofactor PLP are modeled in position equivalent to that of *S*. *cerevisiae* and shown as white stick. The color representation of each domain is same as done for Fig 1A, and the residues present at the active sites are shown as yellow stick. The inhibitor (pink) and cofactor (light orange) are shown for the enzymes from *S*. *cerevisiae* and *G*. *gallus*. The interactions are represented as blue dashed lines.

The active site of *Sp*AST is composed of Tyr70, Ser107, Gly108, Thr109, and Arg263 residues which recognize the phosphate group of PLP; Asn191, Asp219, Tyr222, and Lys255 for interacting with the pyridine ring of PLP; Asn191, Arg289, and Arg383 are involved in identifying the substrate (Fig 2A). When the active site residues of *Sp*AST were compared with either *S*. *cerevisiae* (*Sc*AST) or chicken cytosolic (*Gg*AST) enzyme, most of the side chains, except those of Lys255 and Arg289, were found to have similar conformations. The residues corresponding to Lys258 and Arg292 of *S*. *cerevisiae* and chicken are shown to interact with their cofactor PLP and the inhibitor maleate, respectively (Fig 2B and 2C). The guanidinium group of Arg292 forms a hydrogen bond with the carboxyl group of maleate, and the ε–amino group of Lys258 forms an internal aldimine with the pyridine ring moiety of PLP. Notably, the electron densities of Arg289 and Lys255 in *Sp*AST were somewhat poor, but unambiguously, the positions of Arg289 and Lys255 side chains were located away from the cofactor or inhibitor from *Sp*AST (Fig 2A). This was presumably owing to the absence of its substrate or inhibitor.

A clear density that could be fitted in most likely a phosphate was found in the active site of *Sp*AST (Fig 1D). It might be derived from the protein expression and purification, while there was no ligand added in the crystallization process. Interestingly, the phosphate was located in the corresponding position of the phosphate moiety of PLP (Fig 1F). The phosphate moiety interacts with the main chain of Gly108 and Thr109 and the side chain of Arg263 and Tyr70. The mainchain carboxyl group of Tyr260 interacts NH2 of Arg263. Therefore, it seems that the Tyr260 contributes catalytic activity by stabilizing the conformation of active site, notably Arg263. Tyr260 also forms T-shaped interaction with Tyr70, contributing stabilization of the active site and also dimeric interaction. These features are also found from other homologous structures such as AST from chicken and *S*. *cerevisiae*, indicating it is conserved.

### Domain rotation and conformational change

**Fig 3:**
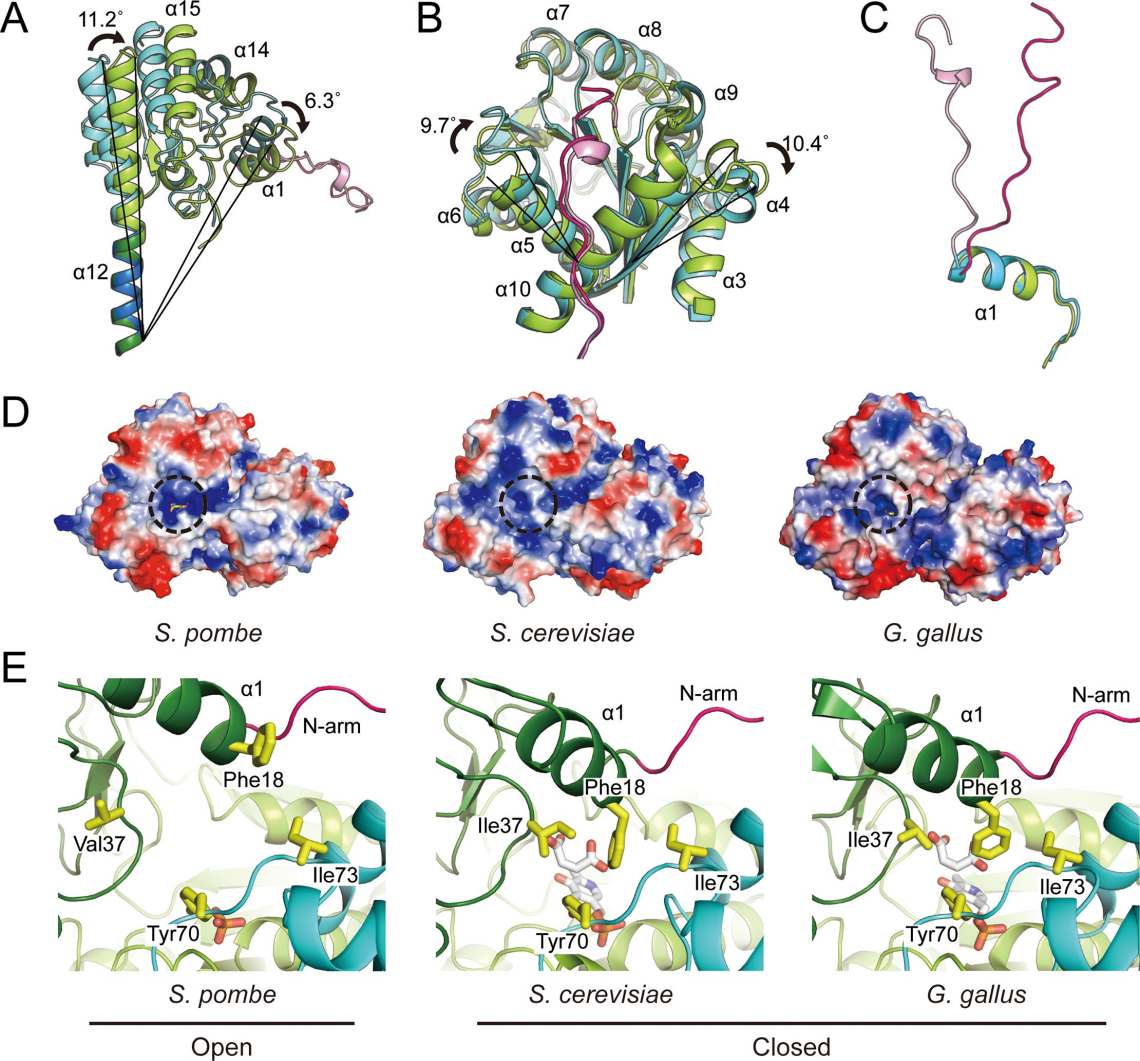
The open–close conformational change of aspartate aminotransferase. The crystal structures of AST from *S*. *pombe* (cyan and pink) and *S*. *cerevisiae* (green and hot pink) were superimposed through the bottom of helix α12 (residues 310–322) for facilitating the comparison between their conformational changes. The three individual segments of each subunit are represented as follows: (A) the small domain shows rigid body rotation tilted from the boundary between the small and large domain; (B) the large domain shows a subtle conformational difference between the two structures; and (C) two structures are superimposed through their α1 helices for aiding the comparison between their N-terminal arms. (D) The structures of the cavities (dashed circle) in ASTs from *S*. *pombe* (left), *S*. *cerevisiae* (middle), and *G*. *gallus* (right) are represented via surface charges. The inhibitor maleate was modeled in the structure of *S*. *pombe* by superimposing with that of *S*. *cerevisiae*. (E) Hydrophobic inter-subunit interaction between α1 of the small domain and the large domain of a partner subunit are shown. Color representation is same as that of Fig 1A. Both the inhibitor and cofactor are represented as white stick, and the phosphate is shown as orange stick.

The ASTs are known to adopt two major conformations “open” and “closed”, upon binding or dissociation of its substrate in its active site, respectively [5, 19]. We next compared the conformational differences of *Sp*AST with the structures of *Sc*AST and *Gg*AST, which adopt a closed conformation. There was a prominent structural transition in each segment of the subunit (Fig 3A–3E). The prominently distinguishable structural differences were observed in the small domain and N-terminal arm, while the large domain showed subtle discrete conformations. Both *Sp*AST and *Sc*AST structures were superimposed with the bottom of the helix α12 (residues 310–322), which spans both large and small domains for the comparison. The helix α12 of *Sp*AST was tilted 11.2° away from central core of the dimer (Fig 3A). The transition makes secondary structural differences between open and closed form of AST, enlarging the diameter of positively charged cavity formed at the area of the active site by 2 Å than that of *Sc*AST (Fig 3D). The diameter of the cavity is 6.7 Å, which allows sufficient entry of its substrate or cofactor; however, a cavity diameter of 4.7 Å, is demonstrated for the *Sc*AST structure. In the closed conformation, the side chain of Phe18 in the helix α1 is packed with hydrophobic residues, such as Ile37, Tyr70*, and Ile73*, at the subunit interface near the active site, possibly forming pi interaction with Tyr70* (Fig 3E) (Asterisk indicates the residues of the adjacent subunit). However, in an open conformation, the hydrophobic interactions were broken because Phe18 showed a different rotameric orientation. Therefore, Phe18 could possibly contribute as a latch to bind as well as to stabilize the substrate or inhibitor at the active site.

The large domain showed a subtle conformational transition (Fig 3B). The central β-sheets and surrounding α-helices were well superimposed, whereas helices α4, α5, and α6 showed clear structural transitions. These helices are located neither at the subunit interface nor at the active site but are present in the vicinity of the N-terminal arm-binding region of the other subunit. Hence, the transition may be possibly induced by the rotation of the small domain and mediated by the N-terminal arm. Thus, the N-terminal arm seems important for the inter-communication between small and large domains upon substrate binding. Therefore, we further compared the positional change of the N-terminal arm (Fig 3C). When the structures were superimposed by the helix α1, the N-terminal arm showed a great extent of rotation at 19.4°. However, when the structures were superimposed by the core of their large domains, both arms were found to be in similar positions, while the helix α1 exhibited a displacement of approximately 3.5 Å. This may indicate that the core of the small domain mainly exhibits structural transition upon enzymatic reaction, while the large domain functions as a scaffold for the dimeric structure and the N-terminal arm acts as a mediator for intercommunication between two subunits.

### Structural classification of PLP-dependent enzyme family

**Fig 4:**
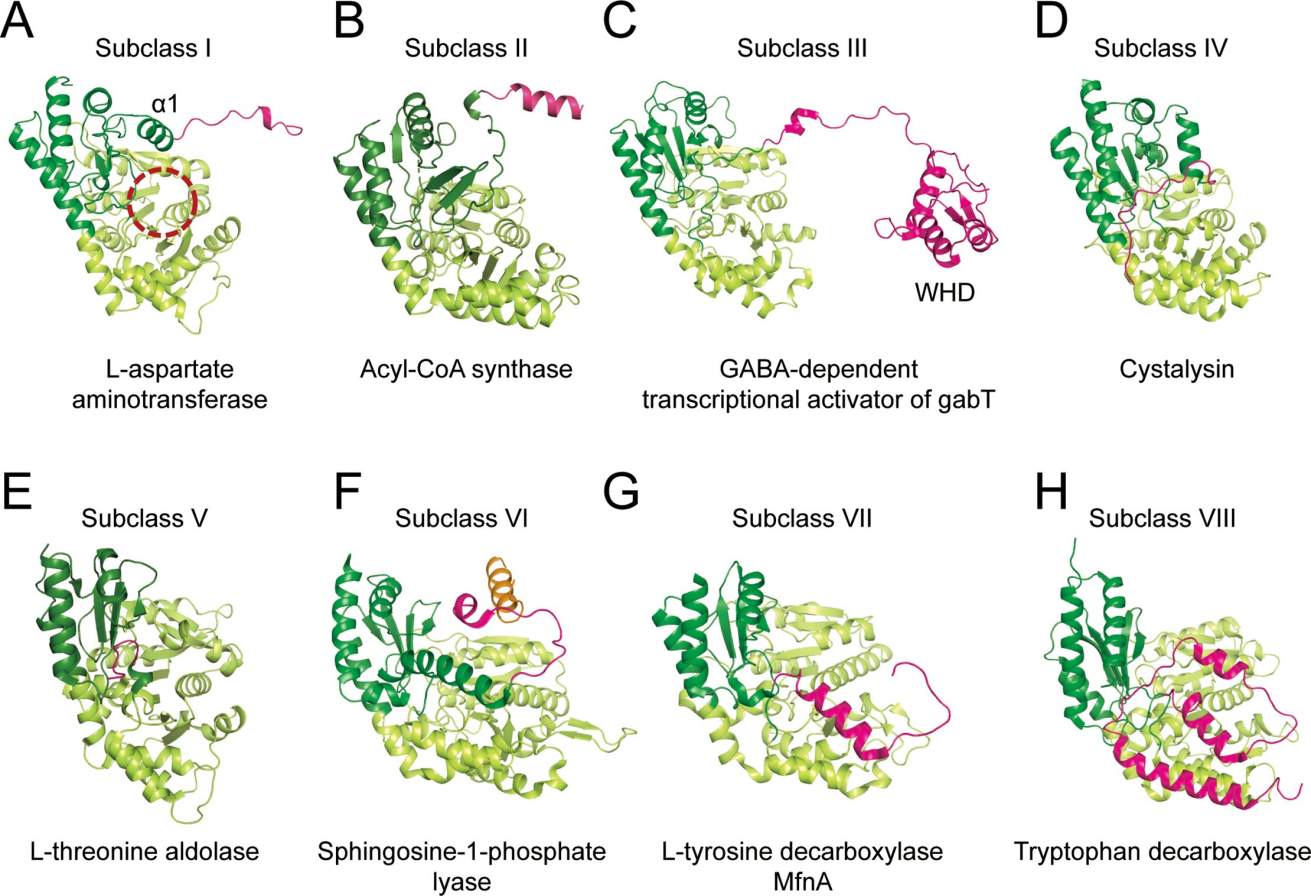
Structural comparison between N-terminal regions. (A-H) Single-subunit structures from homodimeric enzymes are represented and colored as done in Fig 1A. (F) For the type VI enzyme, an additional C-terminal helix was represented in orange. The representative structures for each type are as follows: *Sp*AST for type I; PLP-dependent acyl-CoA synthase (PDB code, 1BS0) for type II; GABA-dependent transcriptional activator of gabT (PDB code, 4MGR) for type III; cystalysin (PDB code, 1C7N) for type IV; L-threonine-O-3-phosphate decarboxylase (PDB code, 1LC5) for type V; sphingosine-1-phosphate lyase (PDB code, 5K1R) for type VI; L-tyrosine decarboxylase MfnA (PDB code, 3F9T) for type VII; Tryptophan decarboxylase (PDB code, 4OBU) for type VIII.

To search the homologous structures of *Sp*AST, web-based DALI server was employed. Approximately two hundred structures were suggested by alignment through their small and large domains. Based on our careful analysis, most structures showed a representative feature of PLP-dependent enzyme family. Prominent differences were observed from their N-terminal region, which is an equivalent region of the N-terminal arm in the *Sp*AST structure, which have also been observed by Lindqvist group [17, 18]. According to the structural characteristics of the N-terminal region, we established eight subclasses (I to VIII) based on the analysis of 71 structures of AST homologs (Fig 4A–4H). The detailed subclass information is listed in Table 2 and S1 Table.

**Table 2:** The subclass classification of selected PLP-dependent enzymes searched by DALI using *Sp*AST as a template.

Enzyme	Species**		Z-score	RMSD (Å)	Identity (%)	Cα	PDB
**Subclass I**							
Aromatic amino acid transferase	*S*. *mutans serotype c*		32.1	3.2	16.0	390	4MY5
1-aminocyclopropane-1-carboxylate synthase	*M*. *domestica*		28.4	3.1	13.1	421	1M4N
Mitochondrial aspartate aminotransferase	*M*. *musculus*		60.3	1.1	42	401	3PDB
**Subclass II**							
PLP-Dependent acyl-CoA synthase*	*E*. *coli*		23.4	4.1	10.4	383	1BS0
5-Aminolevulinate Synthase	*R*. *capsulatus*		22.1	4.5	11.7	396	2BWN
CAI-1 autoinducer synthase	*V*. *cholerae serotype O1*		23.8	4.2	13	329	3KKI
**Subclass III**							
HTH-type transcriptional regulatory protein GabR*	*B*. *subtilis*		27.1	3.7	14.3	468	4MGR
**Subclass IV**							
CYSTALYSIN*	*T*. *denticola*		30.5	3.2	10.5	394	1C7N
Putative pyridoxal phosphate-dependent transferase	*P*. *difficile*		30.0	3.3	13	387	4DGT
Cystathionine beta-lyase	*S*. *anginosus*		29.4	3.5	13.6	388	3B1E
**Subclass V**							
L-threonine aldolase*	*P*. *putida*		21.7	3.6	11.4	343	5VYE
PLP-dependent L-arginine hydroxylase MppP	*S*. *wadayamensis*		26.9	3.9	10.8	369	6C92
L-aspartate beta-decarboxylase	*C*. *testosteroni*		25.7	3.0	15.7	509	2ZY4
**Subclass VI**							
Sphingosine-1-phosphate lyase 1*	*B*. *pseudomallei*		23.4	4.8	8.5	439	5K1R
Sphingosine-1-phosphate lyase 1	*H*. *sapiens*		22.4	4.8	6.0	443	4Q6R
sphingosine-1-phosphate lyase	*S*. *thermophilum*		23.2	4.9	10	441	3MAF
**Subclass VII**							
L-tyrosine decarboxylase MfnA*	*M*. *jannaschii*		22.8	4.5	11.5	394	3F9T
Glutamate decarboxylase	*L*. *brevis*		22.3	5.4	9.8	441	5GP4
Glutamate decarboxylase beta	*E*. *coli*		21.9	5.0	10.8	450	1PMM
**Subclass VIII**							
Pyridoxal-dependent decarboxylase domain protein*	*R*. *gnavus*		22.0	4.2	10.2	462	4OBU
DOPA decarboxylase	*S*. *scrofa*		21.4	4.0	10.7	464	1JS6
Tyrosine decarboxylase 1	*A*. *thaliana*		21.1	4.1	8.6	467	6EEI

* A structure presented in Fig 4.

** The full names of the species are as follows: *S*. *mutans*, *Streptococcus mutans*; *M*. *domestica*, *Malus domestica*; *M*. *musculus*, *Mus musculus*; *E*. *coli*, *Escherichia coli*; *R*. *capsulatus*, *Rhodobacter capsulatus*; *V*. *cholerae serotype O1*, *Vibrio cholera serotype O1*; *B*. *subtilis*, *Bacillus subtilis*; *T*. *denticola*, *Treponema denticola*; *P*. *difficile*, *Peptoclostridium difficile*; *S*. *anginosus*, *Streptococcus anginosus*; *P*. *putida*, *Pseudomonas putida*; *S*. *wadayamensis*, *Streptomyces wadayamensis*; *C*. *testosteroni*, *Comamonas testosteroni*; *B*. *pseudomallei*, *Burkholderia pseudomallei*; *H*. *sapiens*, *Homo sapiens*; *S*. *thermophilum*, *Symbiobacterium thermophilum*; *M*. *jannaschii*, *Methanocaldococcus jannaschii*; *L*. *brevis*, *Lactobacillus brevis*; *R*. *gnavus*, *Ruminococcus gnavus*; *S*. *scrofa*, *Sus scrofa*; *A*. *thaliana*, *Arabidopsis thaliana*.

Most of the aminotransferases including *Sp*AST fall into subclass I. The helix α1 in the small domain is located on top of the inter-domain interface, closing the active site cavity upon substrate binding (Fig 4A). The N-terminal arm protrudes from the helix α1 and reaches to the backside of the large domain of the subunit opposite to it. In the structure of α-aminoadipate aminotransferase from *Thermus thermophilus* (PDB code, 2ZP7), α1 makes two salt bridges with loop α5-β2 and α10 in the same subunit. The region of N-terminal arm forms helical structure and contact large domain of the partner subunit [30]. These features may support dimeric interaction more stable, possibly reinforcing the inter-communication between two subunits upon conformational change.

The two antiparallel β-sheets, instead of helix α1, were observed over the active site in subclass II enzymes (Fig 4B). Although their conformation is not changed upon substrate binding, the helix α14 and loop α14-β9 undergo conformational change from open to closed to adopt its substrate [31]. A three turned helix was observed at the N-terminus which may supports the role of N-terminal arm in the subclass II enzymes (Fig 4B).

Transcriptional activator of GabT (GabR) from *Bacillus subtilis* (PDB code 4MGR) was classified as subclass III and showed the most striking feature among the subclasses. The subclass III showed that the N-terminal region was further extended toward the large domain of the opposite subunit and formed a winged-helix DNA-binding domain (WHD) (Fig 4C). As a result, additional subunit interface was formed between N-terminal WHD and the large domain of opposite subunit.

The subclass IV showed domain folds similar to subclass I, except for helix α1 and N-terminal arm. The helix α1 rotated almost perpendicular to that of subclass I, and the N-terminal arm followed a completely different path (Fig 4D). Notably, the N-terminal arm rotated backward and interacted with the small domain of the same polypeptide chain and not with that of another subunit. In the cystalysin from *T*. *denticola* (PDB code, 1C7N) structure, the Lys8 of N-terminal arm makes hydrogen bond and salt bridge of the main chain of Glu32 on loop α1-α2, and side chain of Glu381 in helix α15, respectively [32]. These interactions make the cavity at the active site further tightly closed. Cystalysin structure showed little conformational change upon substrate binding, with the root mean square deviation of 0.17 Å.

The structures belonging to subclass V contained relatively short N-terminal arm followed by helix α1, which could not contribute to dimerization interface (Fig 4E). Exceptionally, for the L-threonine aldolase from *Pseudomonas putida* (PDB code, 5VYE), even helix α1 is missing and replaced by short loop, leaving the cavity of active site widely open. But this is not a common feature of subclass V.

The subclass VI has unique feature on its small domain as well as N-terminal arm in sphingosine-1-phosphate lyase 1 structure from *Burkholderia pseudomallei* (PDB code, 5K1R). The helix α1 was extended by three turns and reached to the large domain of opposite subunit (Fig 4F). The N-terminal arm, together with the extended α1 helix, formed a helical bundle with the helices in large domain of opposite subunit, which seemed to enhance inter-subunit interaction. However, it seemed not to close the cavity for substrate trapping. This subclass enzyme contained an additional helix at the C-terminal region and wrapped a core beta-sheets of large domain in the opposite subunit. The accessibility to the active site was not perturbed by either the N-terminal arm nor the C-terminal helix, suggesting that they might not affect open to close conformational change. The structural comparisons between apo form and substrate bound form of cystalysin (PDB codes, 1C7N and 1C7O), L-threonine-O-3-phosphate decarboxylase (PDB codes, 1LC7 and 1LC5), and the sphingosine-1-phosphate lyase (PDB codes, 3MAU and 3MBB) from subclasses IV, V, and VI, respectively have shown that these subclasses of family didn’t undergo domain-domain rotation upon substrate binding. The N-terminal arms of these subclasses are either short in length or somewhat displaced from its partner subunit. This further supports that the N-terminal arm might be involved in the inter-subunit communication upon substrate binding, regulating the conformational change during enzymatic reaction. Apparently, the absence of inter-communication between two subunits through the N-terminal arm in class IV enzyme may be also possibly resulted in the lack of open-close conformational transition.

The structures belonged to both subclasses VII and VIII shared similar structural feature on its N-terminal arm, forming a helical bundle with the helices in large domain of the opposite subunit (Fig 4G and 4H). Tryptophan decarboxylase from *Ruminococcus gnavus* (PDB codes, 4OBU and 4OBV) showed no rigid body rotation on the small domain upon substrate binding, but clearly showed subunit rotation between monomers in homodimer [33]. The major difference was observed in the conformation of an extended loop (residues 337–349) in the large domain. Upon substrate binding, this loop rotates and forms a salt bridge and a hydrogen bond to the core β-sheet of small domain in the partner subunit. Although the N-terminal region is displaced from its classical position, this loop replaces the function of the N-terminal region and closes the cavity near active site. The L-tyrosine decarboxylase MfnA from *Methanocaldococcus jannaschii* (PDB code 3F9T) of subclass VII also contained this loop, which indicates that two subclasses are closely related.

The N-terminal arms of subclass III and VIII form extra fold which might be involved in further oligomerization as seen in the structure of gabR of subclass III (PDB code 4MGR), in agreement with the report of Lindqvist and co-workers [17]. Interestingly, the rigid body rotation in the small domain and tilt in the helix α12 were not appeared upon PLP binding in subclass III [34]. Instead, the remarkable rigid body rotation with a transition in average 7Å was shown in both WHDs of each subunit. This inter-subunit transition may introduce closing the cavity at the active site by forming hydrogen bond between Asp144 and Arg451 from the other subunit of homodimer. These features comprehensively emphasizes the importance of N-terminal arm in oligomerization and conformational transition during enzymatic reaction. The diverged feature of N-terminal arms among the PLP-dependent enzymes implicates that the catalytic strategy employed by this cofactor has been diverged during earlier occasions of evolutionary process, although the mode of substrate recognition is well conserved.

### Evolutionary relationship between subclasses

**Fig 5:**
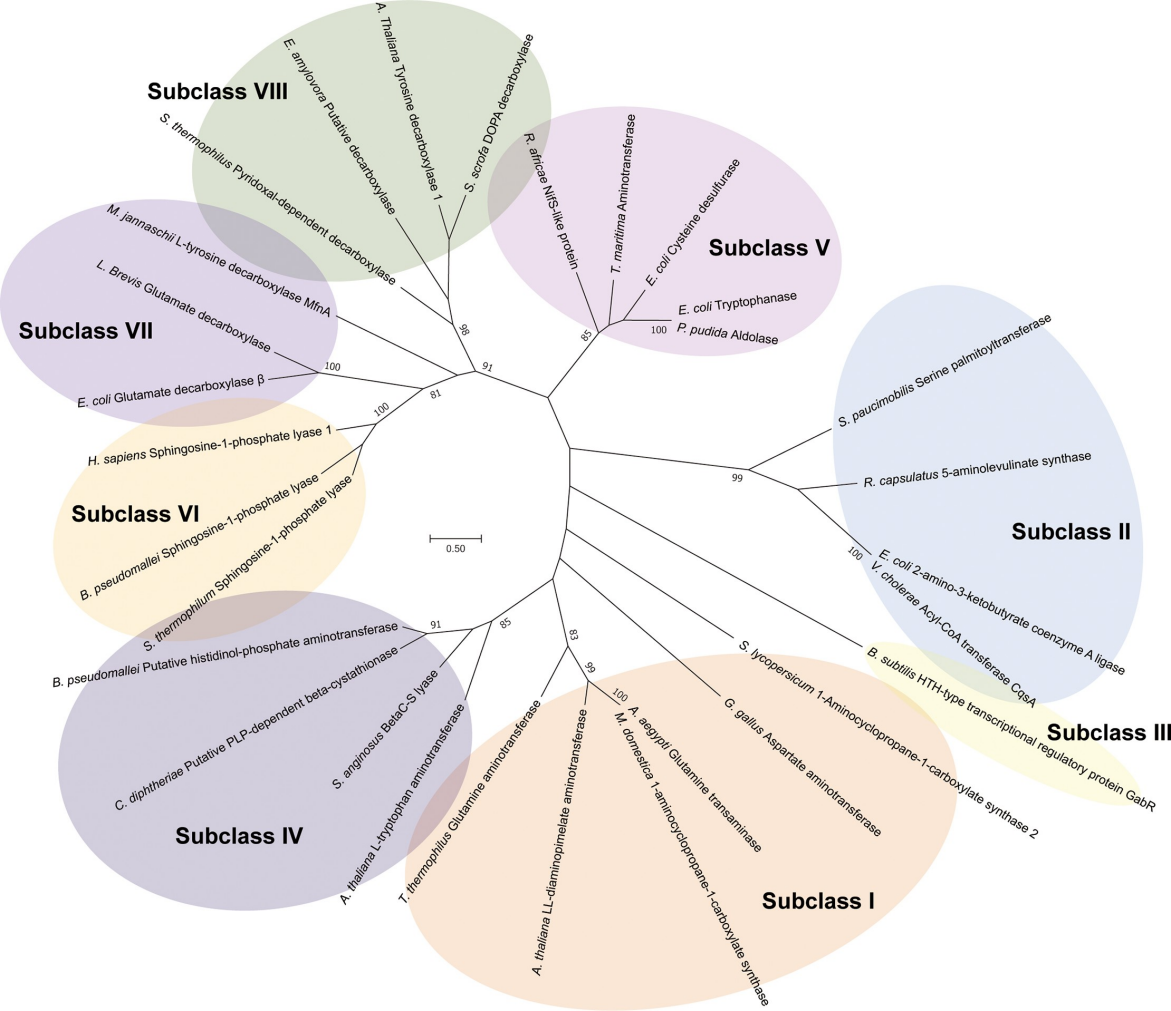
Phylogenetic tree of the PLP-dependent enzyme subclasses. The phylogenetic tree inferred from the 30 PLP-dependent enzymes covering 8 subclasses with bootstrap percentages for 100 replicates. Sequence alignment was carried out using Clustal X, followed by construction of phylogenetic tree using Mega 7. No outgroup was included due to the large divergence in this study. Bootstrap values under 70% are not shown. The full names of the genera with species are shown in S1 Table with abbreviations. Each of the subclasses are presented by discrete colors and indicated. The scale bar indicates the branch length.

To assess the correlation between structure and sequence of the eight subclasses, phylogenetic analysis was performed with 29 out of 71 analyzed PLP-dependent enzymes (S1 Table). The 29 proteins were selected from each of the subclasses which showed relatively low z-scores when their structures were aligned to that of *Sp*AST. The phylogenetic tree data were generated using Maximum-Likelihood method (Fig 5). Interestingly, the taxa were mostly arranged according to the subclasses. Three main clades were shown in the phylogenetic tree. The first clade included subclasses I, III, and IV. The second clade contained subclasses VI, VII, and VIII, while the third clade covered subclasses II and V. A detailed view of the phylogenetic tree presented that GabR in the subclass III was branched out at early stage of the first clade may be due to its discrete sequence and an additional WHD domain. The subclass VI, VII, and VIII showed relatively well distributed in the second clade that may indicate their functional relevance. In addition, they all shared similar features having additional domain or motif at the N-terminal region as described in the previous section. The subclasses I and IV are also closely related as classified as the first clade. This was also predictable since they showed difference only in orientation of the N-terminal arm. However, unexpectedly, it turns out that the subclass V is branched out at the early stage of the evolutionary process and rather related to the subclass II. The subclass II showed similar feature of N-terminal arm compared to subclass I, but the lack of helix α1 and insertion of β sheet in the small domain might be a critical feature that differentiate those from subclass I.

Overall, both the structural classification and phylogenetic tree based on the sequence indicate that the N-terminal features of PLP-dependent proteins have been differentiated during the evolutionary process resulting in different specificity of the substrate or enzymatic mechanism.

## Conclusion

We determined the three-dimensional crystal structure of *Sp*AST and compared to the structures of homologous species. Based on the results, we observed the mode of intercommunication during catalytic reactions between two protomers of the dimer. Extensive comparison between homologous structures and their sequences emerged a novel classification as the PLP-dependent enzyme family have eight subclasses. Our results may provide insights into understanding the diverged enzymatic mechanism of this family.

## Protein data bank accession code

The atomic coordinate and structure factor have been deposited at the Protein Data Bank, with an accession code 6JPK.

## Supporting information

S1 Table List of homologous structures analyzed for enzyme classification. (XLSX)

## Abbreviations

PLP: Pyridoxal 5ʹ-phosphate
AST: L-aspartate aminotransferase
*Sp*: *Schizosaccharomyces pombe*
*Sc*: *Saccharomyces cerevisiae*
*Gg*: *Gallus gallus*
DTT: Dithiothreitol
